# DIA-based technology explores hub pathways and biomarkers of neurological recovery in ischemic stroke after rehabilitation

**DOI:** 10.3389/fneur.2023.1079977

**Published:** 2023-03-07

**Authors:** Wei Hu, Ping Li, Nianju Zeng, Sheng Tan

**Affiliations:** ^1^Department of Neurology, Zhujiang Hospital, Southern Medical University, Guangzhou, China; ^2^Department of Rehabilitation, Xiangya Bo'ai Rehabilitation Hospital, Changsha, China

**Keywords:** ischemic stroke, rehabilitation, data-independent acquisition, parallel response monitoring, biomarkers, bioinformatics

## Abstract

**Objective:**

Ischemic stroke (IS) is a common disease that causes severe and long-term neurological disability in people worldwide. Although rehabilitation is indispensable to promote neurological recovery in ischemic stroke, it is limited to providing a timely and efficient reference for developing and adjusting treatment strategies because neurological assessment after stroke treatment is mostly performed using scales and imaging. Therefore, there is an urgent need to find biomarkers that can help us evaluate and optimize the treatment plan.

**Methods:**

We used data-independent acquisition (DIA) technology to screen differentially expressed proteins (DEPs) before and after ischemic stroke rehabilitation treatment, and then performed Gene Ontology (GO) and pathway enrichment analysis of DEPs using bioinformatics tools such as KEGG pathway and Reactome. In addition, the protein–protein interaction (PPI) network and modularity analysis of DEPs were integrated to identify the hub proteins (genes) and hub signaling pathways for neurological recovery in ischemic stroke. PRM-targeted proteomics was also used to validate some of the screened proteins of interest.

**Results:**

Analyzing the serum protein expression profiles before and after rehabilitation, we identified 22 DEPs that were upregulated and downregulated each. Through GO and pathway enrichment analysis and subsequent PPI network analysis constructed using STRING data and subsequent Cytoscape MCODE analysis, we identified that complement-related pathways, lipoprotein-related functions and effects, thrombosis and hemostasis, coronavirus disease (COVID-19), and inflammatory and immune pathways are the major pathways involved in the improvement of neurological function after stroke rehabilitation.

**Conclusion:**

Complement-related pathways, lipoprotein-related functions and effects, thrombosis and hemostasis, coronavirus disease (COVID-19), and inflammation and immunity pathways are not only key pathways in the pathogenesis of ischemic stroke but also the main pathways of action of rehabilitation therapy. In addition, IGHA1, LRG1, IGHV3-64D, and CP are upregulated in patients with ischemic stroke and downregulated after rehabilitation, which may be used as biomarkers to monitor neurological impairment and recovery after stroke.

## 1. Introduction

Ischemic stroke is a common group of diseases that cause functional impairment and severely affect the patient's ability to perform daily life due to its highly disabling nature. Post-stroke rehabilitation is essential to restore the corresponding deficit function as soon as possible. However, as the assessment of neurological recovery after stroke rehabilitation is mainly performed using scales and imaging, it is difficult to provide a timely reference for the development of efficient treatment strategies. Therefore, there is an urgent need to find appropriate biomarkers that can provide valuable information to neurologists and rehabilitation physicians, which can help optimize the treatment plan and can even help find another treatment modality that can benefit patients when they are not sensitive to one treatment method. Given the heterogeneity of ischemic stroke, biomarkers may provide support for developing the field of ischemic stroke rehabilitation and treatment in clinical settings. In this study, we used data-independent acquisition (DIA) proteomics technology to detect differences in serum protein expression before and after treatment in patients with ischemic stroke. Subsequently, advanced Gene Ontology (GO) and pathway enrichment analysis of differentially expressed proteins (DEPs) were performed using bioinformatics tools such as the KEGG pathway, Reactome, and PANTHER. In addition, we identified the hub proteins (genes) and key signaling pathways involved in rehabilitation therapy for neurological recovery by integrating the protein–protein interaction (PPI) network (http://string-db.org) and modularity analysis of DEPs. Based on this, we selected a portion of the proteins of interest for validation using parallel response monitoring (PRM). The identification of DEPs and the enrichment analysis of their biological functions and key pathways were performed to screen for future biomarkers that may be useful for monitoring neurological recovery after ischemic stroke, which may assist in assessing neurological function and improving treatment strategies.

## 2. Materials and methods

### 2.1. Sample information

Blood samples for this study were collected from patients with stroke rehabilitation admitted to the hospital between June 2020 and December 2020. First, screening was performed according to inclusion and exclusion criteria. Inclusion criteria: (a) patients aged ≥18 years who met the diagnostic criteria for ischemic stroke in the Diagnostic Key Points of Various Major Cerebrovascular Diseases in China (2019) and confirmed by imaging techniques; (b) those with neurological dysfunction (NIHSS score ≥1) with a disease duration of <3 months and stable condition; and (c) those who signed informed consent. Exclusion criteria: (a) patients with severe cardiac insufficiency; (b) those with hepatic and renal impairment (ALT, AST, BUN, and Cr ≥ 2 times the high limit of normal); (c) patients with psychiatric disorders, advanced tumors, and hematological diseases; (d) pregnant and lactating women; and (e) those who withdrew from the study for any reason during the study. Calculation of sample size was performed by PASS 15 (version: 15.0.5) Power Analysis and Sample Size Software (2017), with α = 0.05, 1 – β = 0.9, and dropout rates=20% (NCSS, LLC. Kaysville, Utah, United States, ncss. com/software/pass). Based on the standard clinical treatment, hemiplegic limb function training was given once a day for 30 min for 30 days, and for the presence of speech dysfunction, cognitive dysfunction, or swallowing dysfunction, appropriate rehabilitation treatment was given. To reduce the bias in selecting the study sample, we used the random number table method to randomly select four cases from the 60 total cases for observation. Peripheral blood samples were collected before and after 30 days of rehabilitation treatment. After centrifugation at 3,000 r/min for 15 min, sera were separated and stored in a refrigerator at −80°C for further testing. In addition, to reduce the effect of confounding factors such as age, sex, and race, we matched the selection of healthy controls accordingly. Information on the control group before and after treatment of patients with ischemic stroke is shown in Table 1. This study was supported by the Provincial Department of Science and Technology.

**Table 1:** Sample numbers and groupings.

**Sample number**	**CI-1**	**CI-2**	**CI-3**	**CI-4**	**RT-1**	**RT-2**	**RT-3**	**RT-4**
Group	CI	CI	CI	CI	RT	RT	RT	RT

### 2.2. Instruments and reagents

#### 2.2.1. Main instrumentation

The main instruments used in this experiment were a Q Exactive HF mass spectrometer (Thermo Fisher Scientific, USA), electronic balance (Yue Ping, Shanghai), EASY-nLC 1,000 liquid chromatograph (Thermo Fisher Scientific, USA), enzyme marker (Kehua, Shanghai), TGL-16A benchtop freezing centrifuge (Luxiang Yi, Shanghai), SDS-PAGE gel electrophoresis instrument (Liuyi, Beijing), Tanon 1600 gel imager (Shanghai Tennant), lyophilizer (Ningbo Scientz), certain LG protein stainer (Nanjing GenScript), and high-pH separation liquid chromatograph (Agilent, USA).

#### 2.2.2. Main reagents

The main reagents required for this experiment are as follows: iRT standard peptide was purchased from Biognosys; SDS lysis solution was purchased from Beyotime Biotechnology; bicinchoninic acid (BCA) kit, mass spectrometry grade water, and acetonitrile were purchased from ThermoScientific; performed gum was purchased from GenScript Inc. PMSF was purchased from Amresco; disodium hydrogen phosphate dodecahydrate, sodium dihydrogen phosphate monohydrate, NaCl, Tris-HCl (pH 6.8; pH 8.8), Tris, Dithiothreitol (DTT), glycerol, bromophenol blue, trifluoroacetic acid (TFA), indole-3-acetic acid (IAA), urea, and triethylammonium borate; triethylamine-boric acid (TEAB) were purchased from Biotech; glycine and SDS were purchased from Sinopharm; trypsin was purchased from Wallys; anhydrous ethanol and isopropanol were purchased from GENERAL-REAGENT; and de-high abundance kit was purchased from MilliPore.

### 2.3. Sample preparation and LC-MS/MS high-resolution mass spectrometry detection

A portion of the prepared total protein solution was subjected to protein concentration determination and SDS-polyacrylamide gel electrophoresis using the BCA method. The other part was first taken to complete the enzymatic digestion by trypsin, and the enzymatically digested peptides were desalted by SOLA™ SPE 96-well plate and subjected to LC-MS/MS high-resolution mass spectrometry. The raw files obtained from the detection were imported into Spectronaut Pulsar software for matching, and the machine signals were transformed into peptide and protein sequence information, and then a library of spectra was built based on the relevant information to complete the subsequent DIA analysis.

### 2.4. Differentially expressed protein screening and GO and biological pathway enrichment analysis

Based on the plausible proteins we obtained, differential proteins were screened according to the following criteria: FoldChange of ≥1.2 and a *P*-value of ≤ 0.05 for upregulated proteins; FoldChange of ≤ 0.833 and a *P*-value of ≤ 0.05 for downregulated proteins. To further understand the biological functional information of the differential proteins, we performed GO and pathway enrichment analysis of the candidate DEPs using multiple online databases. We submitted DEPs to the DAVID online program (https://david.ncifcrf.gov/; version: 6.8) with a *p*-value of < 0.05 as the cut-off criterion. In addition, the PANTHER database (http://www.pantherdb.org), the KEGG database (http://www.genome.jp/kegg), BioCyc (http://biocyc.org), and Reactome (http://www.reactome.org) for GO and pathway analysis. Enriched GO terms were ranked according to *P*-values and displayed as bar graphs; a *P*-value of < 0.05 was considered to be statistically significant.

### 2.5. PRM-targeted proteomic validation

We used parallel reaction monitoring (PRM) targeted proteomics to validate some of the screened proteins of interest to validate our study. First, we screened the differential proteins with no current literature or few expressions with ischemic stroke as representative proteins. Peptide information was collected in data-dependent acquisition (DDA) mode, based on the raw data obtained from mass spectrometry assays and then library search, using the software PD2.2. Then, after identifying the candidate target peptides (selecting proteins with ≥2 peptides), PRM mass spectrometry was performed to validate them, and then Skyline software was used to analyze and complete the quantification of the target proteins. In this study, we strictly followed the SOPs for extraction, quantification, quality control, and subsequent enzymatic digestion and desalting of the sample proteins, and finally completed the PRM assay.

## 3. Experimental results

### 3.1. Clinical characteristics of patients

Of the four enrolled patients with ischemic stroke, one was men and three were women. The age of the patients at enrollment was 60.0 ± 6.0 years and the median duration of illness was 19.5 days, with a range of 3–30 days. The pre-treatment NIHSS score was 8.3 ± 1.3, and the post-rehabilitation NIHSS was 4.8 ± 1.7, as detailed in Table 2.

**Table 2:** Clinical characteristics of four ischemic stroke patients.

**No**.	**Sex**	**Age**	**Disease duration (days)**	**Combined diseases**	**Crisis**	**NIHSS score**	
						**Baseline**	**Post-RT**
1	Male	63	25	Hypertension, type 2 diabetes	N	7	3
2	Female	55	30	Hypertension, hyperlipidemia, chronic bronchitis	N	10	7
3	Female	55	14	–	N	8	4
4	Female	67	3	Hypertension, type 2 diabetes, hyperlipidemia	N	8	5

### 3.2. Protein concentration measurement results

The absorbance and concentration of samples are shown in Table 3.

**Table 3:** Protein concentration measurements in ischemic stroke before (CI, *n* = 4) and after rehabilitation treatment (RT, *n* = 4).

**No**.	**Adsorbance 1**	**Adsorbance 2**	**Adsorbance 3**	**Average absorbance**	**Measurement concentration (ug/uL)**	**Real concentration (ug/uL)**
Cl-3	0.180	0.186	0.180	0.182	0.190	1.90
RT-3	0.187	0.180	0.205	0.191	0.200	2.00
Cl-4	0.150	0.171	0.181	0.167	0.174	1.74
RT-4	0.143	0.162	0.180	0.162	0.168	1.68
Cl-1	0.219	0.216	0.198	0.211	0.222	2.22
RT-1	0.146	0.159	0.161	0.155	0.161	1.61
Cl-2	0.304	0.285	0.282	0.290	0.310	3.10
RT-2	0.060	0.096	0.110	0.089	0.087	0.87

### 3.3. SDS-PAGE results

**Figure 1:**
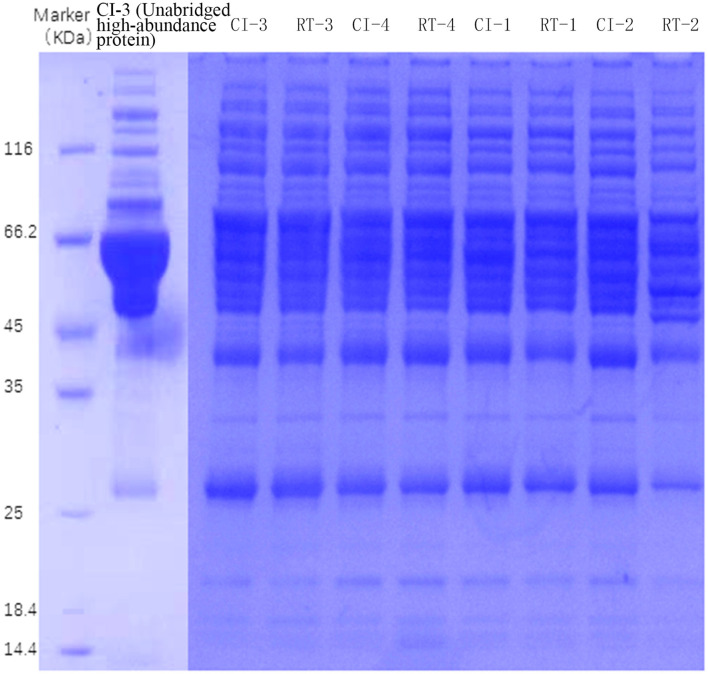
SDS-PAGE electropherogram of samples.

The results of SDS-PAGE are shown in Figure 1.

### 3.4. Trusted protein analysis and data quality control

**Figure 2:**
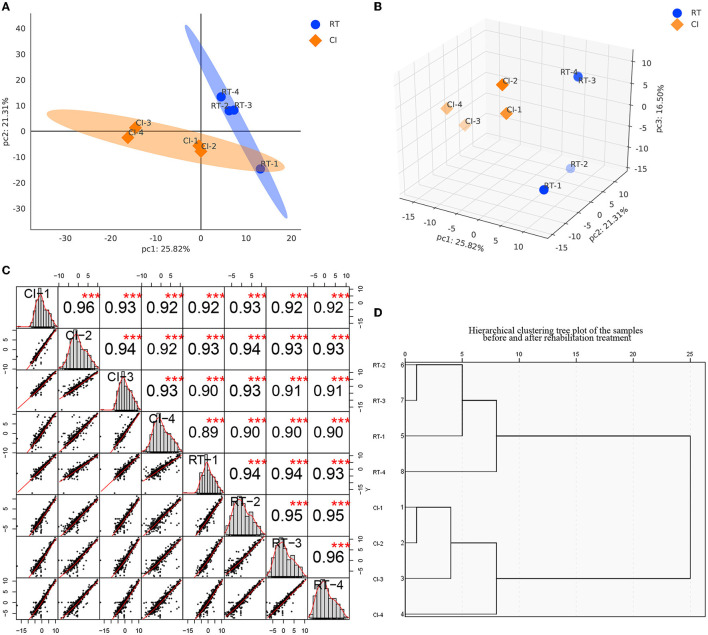
**(A, B)** are the principal component analysis of the expression of plausible proteins before and after rehabilitation treatment [**(A)** is a two-dimensional plot, while **(B)** is a three-dimensional plot]. Each point represents a sample, and the blue represents before treatment, while the orange is after treatment. The more distant the position of the two samples, the more significant the difference is represented. **(C)** Sample Correlation Analysis. The upper triangle (upper right of the diagonal line), the numbers indicate the correlation values of the two samples, * indicates the degree of significance (***p < 0.001); the lower triangle (lower left of the diagonal line), gives the scatter plot of the expression values of the two samples, the red curve is the fitted trend, the greater the slope the stronger the correlation between the two samples; the diagonal line is the expression of the samples themselves distribution graph. **(D)** Sample hierarchical clustering tree diagram. Each branch end represents a sample, and samples clustered within the same branch are considered as samples expressing similar or close features, and samples not clustered into the same branch can be considered as samples with dissimilar or not close features, as measured by the Euclidean distance of the horizontal coordinate.

Based on the raw data obtained, we retained the proteins with expression values of ≥50% for any set of samples, and the proteins with missing values <50% were filled using the k-nearest neighbors (KNN) algorithm (k = 3), followed by median normalization and log2 log transformation, to obtain plausible proteins, and the apparent proteins were visualized demonstration. As shown in Figures 2A, B, we performed principal component analysis (PCA) based on the expression of plausible proteins to show the interrelationship between samples from different dimensions, and the results suggested significant differences between the two groups of proteins before and after rehabilitation treatment and good intra-group reproducibility. In the sample correlation analysis of plausible proteins (Figure 2C), by measuring the degree of correlation between samples, the results showed that the two groups of samples were grouped matching their conditions and had good reproducibility. In addition, sample hierarchical clustering analysis was performed in this study and presented in a clustering dendrogram. As shown in Figure 2D, the classification of the samples before and after the rehabilitation treatment matched the clinical one. We standardized the data to minimize the effect of systematic bias on the quantitative protein values of the samples in this study so that the data obtained from each sample and parallel experiments could be at the same level, thus making the subsequent analysis more accurate and reliable, and the median of the data after standardization tended to be in the center.

### 3.5. Differential protein expression analysis

**Figure 3:**
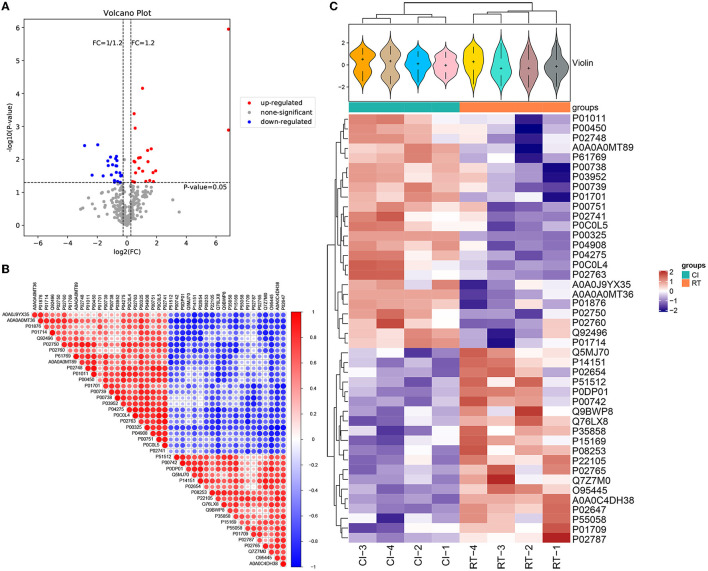
**(A)** Volcano plot of differentially expressed proteins. The horizontal coordinate of the volcano plot is log2 (FC), and its value is farther from the 0 point indicating a larger difference, with upward adjustment on the right and downward adjustment on the left. The vertical coordinate is –log10 (*P*-value), and the farther the value is from the 0 point, the larger the difference is. The blue points in the graph indicate down-regulated differentially expressed proteins, the red points are up-regulated differentially expressed proteins, and the gray points are non-significant differentially expressed proteins. **(B)** Correlation analysis of differentially expressed proteins. In the figure, red is positive correlation, blue is negative correlation, the size of the circle represents the level of significance of the correlation test, and the color shade represents the magnitude of the correlation coefficient. **(C)** Cluster analysis heat map of differentially expressed proteins. The upper part is a violin plot, the violin plot is a combination of box line plot and density plot, the flatter the box the more concentrated the data are, the outline of the box reflects the probability distribution of expression values, different color fills represent different samples, the “+” in the middle of the violin indicates the median of the data, the vertical axis is protein expression; the column annotations of the heat map are below the violin, the same. The samples in the same subgroup correspond to the same color block annotation; the clustering heat map below, clustering according to protein expression, red indicates high expression protein, blue indicates low expression protein, each row indicates the expression of each protein in different groups.

For screening DEPs based on obtaining plausible proteins, we selected both the Foldchange value of the fold change in expression level and the *p*-value of the significance level. In our study, the differential screening criteria for the project were Foldchange = 1.2 and *p*-value of < 0.05, where FC = 0 and FC = inf were both with or without differences. After the screening, as shown in Table 4, we obtained a total of 44 DEPS containing 22 upregulated and 22 downregulated proteins. The overall distribution of DEPS was visualized as shown in Figure 3A (volcano plot). In addition to that, we used R language to perform hierarchical clustering on the normalized data. As shown in Figure 3C, the differences in protein expression between the two groups before and after the rehabilitation treatment were obvious, and the samples within the group also appeared in the same cluster by clustering. Moreover, by using the Pearson analysis to analyze the differential proteins, we obtained the corresponding correlation coefficients, the higher the coefficient, the stronger the correlation between the proteins. To demonstrate the association of differential protein expression, we made a TOP50 differential significant protein (*p*-value ranking) correlation analysis graph, as shown in Figure 3B.

**Table 4:** 44 differentially expressed proteins (DEPs) were identified in the study, including 22 up-regulated proteins and 22 down-regulated proteins in ischemic stroke before and after rehabilitation treatment.

**DEPs**	**Proteins ID**
Down-regulated	A0A0A0MT89, A0A0J9YX35, P00450, P00738, P00739, P00751, P01011, P01701, P01714, P01876, P02741, P02748, P02750, P02760, P02763, P03952, P04275, P04908, P0C0L4, P0C0L5, P61769, Q92496
Up-regulated	A0A0A0MT36, A0A0C4DH38, O95445, P00325, P00742, P01709, P02647, P02654, P02765, P02787, P08253, P0DP01, P14151, P15169, P22105, P35858, P51512, P55058, Q5MJ70, Q76LX8, Q7Z7M0, Q9BWP8

### 3.6. Protein functional analysis

#### 3.6.1. GO functional enrichment analysis of differential proteins before and after rehabilitation treatment

**Figure 4:**
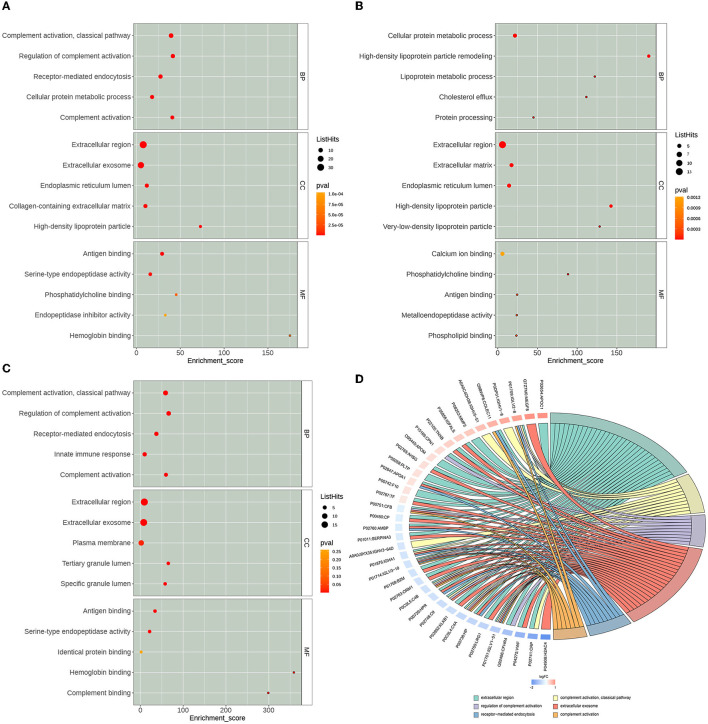
Gene Ontology (GO) enrichment analysis results. The x-axis Enrichment Score in the bubble diagram is the enrichment score, and the y-axis is the top 5 term information of BP, Biological Process; CC, Cell Component; MF, Molecular Function, respectively. **(A)** The top 15 GO terms of all differentially expressed proteins. **(B)** The top 15 GO terms of the up-regulated expressed proteins. **(C)** The top 15 GO terms of the down-regulated expressed proteins. **(D)** GO enrichment analysis chord diagram. Protein:gene name on the left, selected GO term on the right, red indicates up-regulated, and blue indicates down-regulated.

By using multiple online databases for GO enrichment analysis of differential proteins in this study, we obtained 308 Biological Process (BP) categories, 94 Molecular Function (MF) categories, and 64 Cellular Component (CC) categories. As shown in Figure 4, we filtered to the top 15 (five entries each for each category, ranked from largest to smallest by –log10 *P*-value) categories obtained from the GO enrichment analysis. In the BP category, differential proteins were mainly enriched in GO:0006958: Complement activation, classical pathway (List Hits = 11, padj = 3.14E-13), GO:0030449: Regulation of complement activation (List Hits = 9, padj = 6. 87E-11), GO:0006898: Receptor-mediated endocytosis (List Hits = 9, padj =2.19E-09), GO:0006956: Complement activation (List Hits = 7, padj =2.09E-08), and GO:0044267: Cellular protein metabolic processes (List Hits = 8, padj = 6. 44E-07). In the CC category, differential proteins were mainly enriched in GO:0005576: extracellular region (List Hits = 32, padj = 1. 71E-21), GO:0070062: extracellular exosome (List Hits = 24, padj = 8.39E-11), GO:0034364: high-density lipoprotein particle (List Hits = 4, padj = 9.08E-06), GO:0005788: endoplasmic reticulum lumen (List Hits = 8, padj = 1.12E-05), and GO:0034366: spherical high-density lipoprotein particle (List Hits = 3, padj = 1. 88E-05). In the MF category, differential proteins were mainly enriched in GO:0003823: antigen binding (List Hits = 7, padj = 2.03E-07), GO:0004252: serine-type endopeptidase activity (List Hits = 6, padj = 5.60E-05), GO:0031210: phosphatidylcholine binding (List Hits = 3, padj = 0.000526458), GO:0030492: hemoglobin binding (List Hits = 2, padj = 0.000617798), and GO:0055102: lipase inhibitor activity (List Hits = 2, padj = 0.000822536). As shown in Figures 4A–C, we also used bubble plots for the presentation of GO enrichment analysis results. In addition, we also used a chord diagram (Figure 4D) to show the relationship between the GO term and the corresponding differential proteins.

#### 3.6.2. Pathway enrichment analysis of differential proteins

**Figure 5:**
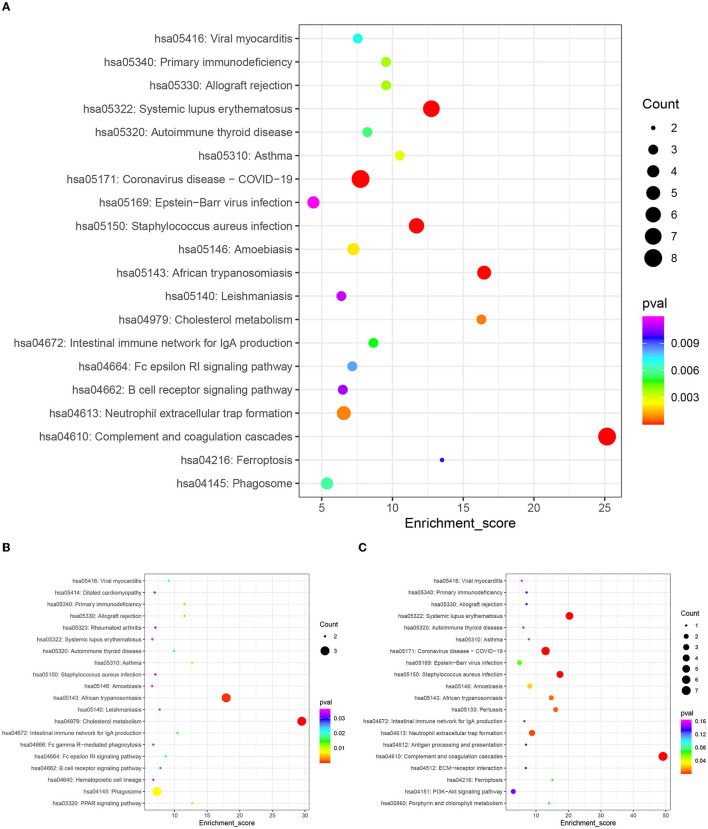
KEGG enrichment top 20 bubble map. The x-axis Enrichment Score is the enrichment score and the y-axis is the pathway information of top 20 in the graph. The larger the bubble the more entries contain the number of differential proteins, the bubble color changes from red-green-blue-purple, and the smaller its enrichment *p*-value value, the greater the significance. **(A)** Top 20 KEGG pathways of all differentially expressed proteins. **(B)** Top 20 KEGG pathways of the up-regulated expressed proteins. **(C)** Top 20 KEGG pathways of the down-regulated expressed proteins.

In this study, we performed pathway enrichment analysis by using the online database of KEGG, and the significance of each enriched pathway was expressed by *p*-value. The *p*-values were calculated using the hypergeometric distribution test. The results are shown in Figure 5. The differential proteins before and after rehabilitation mainly involved hsa04610: complement and coagulation cascade (pval = 5.37E-10, enrichment score=25.1661442), hsa05322: systemic lupus erythematosus (pval = 8.36E-07, enrichment score=12.74863884), hsa05171: coronavirus disease (COVID-19) (pval = 5.26E-06, enrichment score = 7.743428985), hsa05150: *Staphylococcus aureus* infection (pval = 9.36E-06, enrichment score = 11.69694026), hsa05143: African trypanosomiasis (pval = 1.08E-05, enrichment score = 16.47783251), hsa04979: cholesterol metabolism (pval = 0.00078561, enrichment score = 16.28397566), hsa04613: neutrophil extracellular trap formation (pval = 0.000848819, enrichment score = 6.559895408), and other pathways. Among them, the upregulated differential protein pathways were enriched in hsa04979: cholesterol metabolism (pval = 0.000127618, enrichment score = 29.51470588), hsa05143: African trypanosomiasis (pval = 0.000560924, enrichment score = 17.91964286), and hsa04145: phagosomes (pval = 0.007285046, enrichment score = 7.307038835), while downregulated differential protein pathways were enriched in hsa04610: complement and coagulation cascade (pval = 2.43E-11, enrichment score = 49.12237762), hsa05322: systemic lupus erythematosus (pval = 2.59E-06, enrichment score = 20.31376518), hsa05171: coronavirus disease (COVID-19) (pval = 2.70E-06, enrichment score = 12.95535234), hsa05150: *Staphylococcus aureus* infection (pval = 5.93E-05, enrichment score = 17.39544962), and hsa04613: neutrophil extracellular trap formation (pval = 0.004212885, enrichment score = 8.7801677), hsa05133: Pertussis (pval = 0.93E-05, enrichment score = 17.39544962), hsa04613: neutrophil extracellular trap formation (pval = 0.004212885, enrichment score = 8.7801677), hsa05133: pertussis (pval = 0.006614365, enrichment score = 16.03996004), hsa05143: African trypanosomiasis, pval = 0.007830099, enrichment score = 14.7032967), and other pathways.

#### 3.6.3. Differential protein interaction network analysis

**Figure 6:**
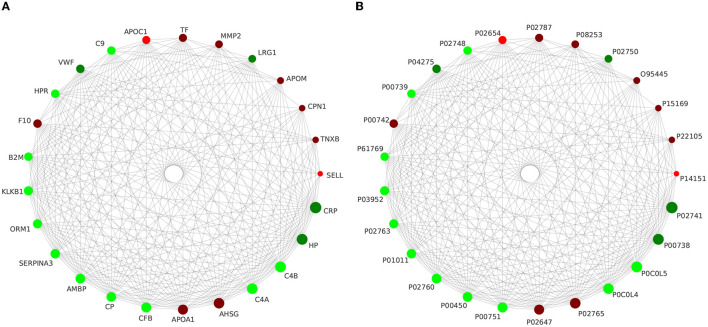
Interaction network analysis of the top 25 differential proteins in terms of connectivity is presented with gene name **(A)** and protein ID **(B)**, respectively.

Using the STRING online database to analyze the differentially expressed proteins in this study, we constructed the interaction network of differential proteins, in which CRP, HP, C4B, C4A, AHSG, APOA1, CFB, CP, AMBP, and SERPINA3 were the 10 proteins with the highest connectivity. In this study, we selected the top 25 proteins in terms of connectivity and used the python package 'networkx 2.1' to draw a protein interaction network map. The top 25 nodes in terms of node connectivity were visualized by the python package 'networkx' and displayed by protein ID and gene name, respectively (as shown in Figure 6).

### 3.7. PRM validation of target peptides

**Figure 7:**
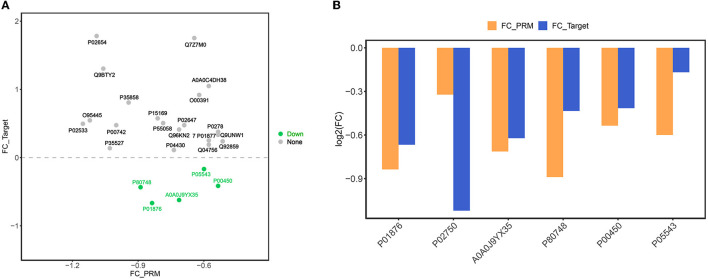
**(A)** Scatter plot of target protein expression distribution. The x-axis is the log2 (FC) value of PRM-identified proteins, and the y-axis is the log2 (FC) value of DIA target proteins. Green dots indicate proteins with down-regulated expression trends of both PRM and DIA, orange dots are proteins with up-regulated expression of both PRM and DIA, and gray dots are proteins with inconsistent expression trends of both. **(B)** Bar chart of target protein expression trend. The x-axis is the ID of the protein with consistent expression trend of PRM and DIA, and the y-axis is the log2 (FC) value of the corresponding protein. log2 (FC) >0 is the protein with up-regulated expression, and log2 (FC) >0 is the protein with down-regulated expression.

By importing the off-board data into SpectroDive software for analysis, we manually corrected the identification of each peptide in each sample after the peak extraction was finished, adjusted the peptides with offset, and derived the quantitative results of the target peptides. In this experiment, we selected 14 proteins that were differentially expressed before and after rehabilitation treatment, namely IGHV5-51, IGHV3-64D, APOM, CP, F10, IGHA1, APOA1, APOC1, LRG1, TF, CPN1, IGFALS, PLTP, and MEGF8, in addition to QSOX1, IGHA2, KRT14, SLC4A1, IGKV1-16, SERPINA7, KRT9, IGLV3-21, HGFAC, CHIT1, OIT3, NEO1, CNDP1, FUCA2, and MINPP1, and 15 proteins of interest that were differentially expressed in ischemic stroke and healthy subjects in the previous studies and which are less frequently or not. These proteins of interest, which are less frequently or not reported in the literature, were directly associated with ischemic stroke. Based on the derived quantitative information of the target peptides, we used the software built-in mean peptide quantity (i.e., mean peptide quantification) to perform the calculation of the quantitative values of the proteins and used them for subsequent statistical analysis between groups. Then, we performed the analysis of the target protein expression values according to the sample comparison groups separately and plotted the scatter plot of the target protein distribution for each comparison group (Figure 7A). Finally, we then performed expression analysis and visualization based on the data of proteins with consistent expression trends in the comparison groups and plotted the histograms of the target proteins with consistent expression trends, as shown in Figure 7B. After PRM validation, six proteins, including IGHA1, LRG1, IGHV3-64D, IGLV3-21, CP, and SERPINA7, were consistently downregulated in expression after rehabilitation treatment.

## 4. Discussion

Ischemic stroke, the most common type of cerebrovascular disease, is characterized by high disability, morbidity, mortality, and recurrence. Although rehabilitation therapy has a good effect on improving neurological function in ischemic stroke, the current assessment of neurological recovery is mainly based on a scale, which is limited by its timeliness and objectivity. Currently, there are two main categories of assessment of neurological recovery, one is the scale category, which is relatively well-established and most widely used. The other category is functional imaging assessment, which is not yet immature, costly, and not widely used. The most commonly used scales are the National Institutes of Health Stroke Scale (NIHSS), the Glasgow Coma Scale (GCS), the Modified Rankin Scale (MRS), and the Barthel index (BI). In addition, there are scales such as the Mini-Mental State Examination (MMSE) and the Montreal Cognitive Assessment (MoCA) for assessing cognitive function, and the Self-Rating Scale for Depression (SDS) and the Self-Rating Scale for Anxiety (SAS) for assessing mental status. The NIHSS is a 15-item neurological function examination scale designed in 1989 (1). The NIHSS is currently the most widely used, reliable, valid, and sensitive neurological function assessment scale worldwide. However, the NIHSS is insensitive to posterior circulation stroke and can also underestimate the severity of right hemisphere stroke (2, 3). The Glasgow Coma Scale (GCS) is used to assess the state of impaired consciousness in stroke, determine the severity of the stroke, and predict the prognosis, and is more appropriate for patients under 55 years of age (4, 5). The Modified Rankin Scale (MRS) is mainly used to evaluate stroke outcomes and contains only six rating scale items, resulting in poor inter-rater reliability (6, 7). The Barthel index (BI) consists of 10 items and has good reliability and validity for both face-to-face and telephone assessments, and is often used as a functional indicator of outcome. However, items such as language, cognition, visual acuity, emotional disturbance, and pain were not included, and there was a “ceiling effect” and a “floor effect” on the BI, with a high proportion of cases having a perfect score and a low score in the acute stage, respectively (8–10). Functional neuroimaging assessment includes positron emission tomography (PET), functional magnetic resonance imaging (fMRI), single-photon emission computed tomography (SPECT), magnetic resonance spectroscopy (MRS), and transcranial magnetic stimulation (TMR). Xenon-133 dynamic SPECT, can-18 PET, and (31)P spectroscopic imaging can observe changes in neurological function as it recovers (11). Functional magnetic resonance imaging (fMRI) can detect changes in brain function after motor recovery in patients with early ischemic stroke (12). A model of magnetic resonance texture analysis (MRTA) based on ADC maps can be used to assess neurological function in patients with unilateral anterior circulation ischemic stroke (13). PET provides insight into improvements in speech function by measuring changes in regional cerebral flooding (rCBF) (14). It is crucial to explore the discovery of biomarkers that can predict or assess neurological recovery on the one hand, and to gain a deeper understanding of the mechanisms associated with rehabilitation therapy, on the other hand, to lay the foundation for exploring optimal treatment plans.

In this study, we compared the proteomic changes before and after ischemic rehabilitation treatment, clarified 44 differentially expressed proteins, and found 22 upregulated and downregulated proteins, respectively. Based on this, the differential proteins were subjected to GO enrichment analysis using a bioinformatics approach. The upregulated and downregulated DEPs were further linked to different processes and pathways based on the functions and signaling pathways identified by the significant enrichment analysis. Then, we also constructed a PPI network of DEPs consisting of 44 nodes and 81 edges through the STRING (Version: 11.5) website (the maximum number of linkage demonstrations was chosen to be no more than 10 linkages, and the minimum required interaction confidence was selected to be high confidence 0.700), which had an average node degree of 3.68, an average local clustering coefficient of 0.532, and a predicted number of edges of 14, while the *p*-value for protein interaction enrichment was <1.0e-16. To obtain more meaningful clusters, we set k = 7. In addition, the four most essential clusters in the PPI network complex, including 17 central node proteins, were screened by Cytoscape (version: 3.9.1) MCODE. Finally, we also validated the 29 proteins of interest using PRM, and the results suggested that among our 29 proteins of interest, PRM validated a total of six proteins including IGHA1, LRG1, IGHV3-64D, IGLV3-21, CP, and SERPINA7 to be consistently downregulated in expression after rehabilitation treatment.

The first protein cluster contains eight proteins including C3, C4A, C4B, CFB, CFHR4, CPN1, CRP, and VWF, which are enriched in hsa04610: complement and coagulation cascade (FDR = 6.48E-11), hsa05150: *Staphylococcus aureus* infection (FDR = 4.85E-06), hsa05133: Pertussis (FDR = 0.00036), hsa05133: pertussis (FDR = 0.00036), and hsa05322: systemic lupus erythematosus (FDR = 0.00053) by the KEGG pathway enrichment analysis. The Reactome pathway enrichment analysis showed that it was mainly enriched in HSA-166658: complement cascade (FDR = 4.93E-14), HSA-977606: regulation of complement cascade (FDR = 8.91E-12), HSA-166663: initial triggering of complement (FDR = 1.36E-10), HSA-174577: activation of C3 and C5 (FDR = 2.05E-09), and HSA-173736: activation of alternative complement (FDR = 0.00095). The WikiPathways enrichment analysis revealed its enrichment in WP2806: complement system (FDR = 3.45E-10), WP545: complement activation (FDR = 5.78E-08), WP558: complement and coagulation cascade (FDR = 1.43E-06), WP5090: complement system in neuronal development and plasticity (FDR = 1.06E-05), WP2328: allograft rejection (FDR = 0.00075), and WP5104: acquired partial steatosis/Barraquer-Simons syndrome (FDR = 0.0011) pathways. Among these proteins, complement C3 is associated with inflammation and ischemia/reperfusion injury and with poor outcomes after ischemic stroke, which may increase its risk within 3 months (15). Brain endothelial cells are susceptible to direct infection by SARS-CoV-2, which causes upregulation of complement component C3 and increases the association of cerebrovascular events after SARS-CoV-2 infection (16). Overexpression of C4A decreases the cortical synaptic density, increases microglia phagocytosis of synapses, and affects neurological function (17). Increased C4B levels reinforce microglia phagocytosis of synapses and synaptic loss in the hippocampal CA3 region (18). Regulation of CFB and other protein levels may attenuate atherogenic tendencies (19). CFHR4 expression levels of immune cell infiltration are closely correlated with the levels of infiltrating DCs, neutrophils, Th17 cells, and mast cells (20). CPN1 may be involved in acute phase response signaling and the development of hypercoagulable and hypofibrinolytic states, and its activation impairs the clearance of impaired mitochondria, leading to mitochondrial dysfunction (21, 22). Elevated plasma CRP, which may increase the risk of ischemic stroke, is closely associated with cryptogenic stroke with systemic inflammatory features and also increases asymptomatic intracranial or extracranial arterial stenosis in patients with AIS or TIA at 1-year risk of ischemic stroke and risk of ischemic stroke recurrence (23–25). VWF interacts with the circulatory system and platelets in hemostasis and thrombosis by sensing and responding to changes in hemodynamics, which have been associated with atherosclerosis, stroke, and more recently, COVID-19 thrombotic symptoms (26).

The second protein cluster has five proteins, ADH1B, GP1BA, KLKB1, KNG1, and TNXB. No significant pathways were obtained from this cluster after the KEGG pathway enrichment analysis, but the Reactome pathway enrichment analysis showed that it was mainly enriched in HSA-140837: Intrinsic pathway of fibrin clot formation (FDR = 4.51E-05), HSA-9651496: contact activation system (CAS) and defective kallikrein/kinin system (KKS) (FDR = 0.0058). The WikiPathways enrichment analysis showed its enrichment in WP4969: RAS and bradykinin pathway of COVID-19 (FDR =0.0164) and WP558: complement and coagulation cascade (FDR = 0.0311). Of these proteins, ADH1B may improve the cognitive function profile of those who drink moderately (27). Genetic variants of GP1BA may be associated with venous thrombosis in Asian ancestry (28). KLKB1 causes a dose-dependent enhancement of the anticoagulant effects of plasma thrombin generation (TG) and coagulation regulatory protein (TM) (29). A decrease in KLKB1 increases the risk of atrial fibrillation (30). However, KLKB1 may increase the risk of cerebral hemorrhagic transformation and angioedema (31). In addition, the KLKB1 gene encodes a procoagulant basic protein, PK, that can regulate circulating cholesterol levels by binding to LDLR and inducing its lysosomal degradation. Blocking PK can stabilize LDLR, lower LDL cholesterol, and thus inhibit further development of atherosclerotic plaques (32). TNXB haploinsufficiency or deficiency may provide some benefit to vascular events such as stroke by attenuating aging-related atherosclerosis and enhancing the body's adaptation to atherosclerotic plaques (33). KNG1 not only plays a central role in coagulation and thrombosis but is also significantly associated with cryptogenic young stroke (34). TNXB plays a role in inhibiting endothelial-to-mesenchymal transition (EndMT) and endothelial inflammation, thus ameliorating atherosclerosis by binding to TGF-beta and blocking its activity (35).

The third protein cluster had a total of seven proteins including COLEC11, F10, IGFALS, MMP16, MMP2, TIMP1, and TIMP2. An enrichment analysis of this protein Reactome pathway showed that it was mainly enriched in HSA-1592389: activation of matrix metalloproteinases (FDR =8.18E-07), HSA-381426: regulation of insulin-like growth factor (IGF)-binding proteins (IGFBPs) on insulin-like growth factor transport and uptake (FDR = 0.005). The WikiPathways enrichment analysis showed its enrichment in WP129: matrix metalloproteinases (FDR = 1.79E-07), WP2865: IL1 and megakaryocytes in obesity (FDR = 0.012), and similarly, the KEGG pathway enrichment analysis of this group of proteins did not enrich for closely related pathways. Among these proteins, COLEC11 belongs to the lectin pathway-related proteins, which have an essential impact on complement-mediated ischemic injury. In addition, the activity of the lectin pathway is reduced in asymptomatic patients with COVID-19 (36, 37). Elevated thrombin levels in the brain further compromise the integrity of the blood–brain barrier (BBB) in patients with stroke, causing direct parenchymal damage. At the same time, systemic F10 inhibition improves neurological outcomes (38). IGFALS is significantly increased in non-valvular atrial fibrillation expression (39), which may increase the risk of ischemic stroke. The downregulation of MMP16 inhibits the migration of vascular smooth muscle cells, which in turn affects the atherosclerotic development process (40, 41). The secretion of MMP2, which promotes cell invasion and migration of vascular smooth muscle cells (VSMCs), influences the process of atherosclerosis (42). Transient inhibition of MMP2/9 after stroke rescues the plasticity of the damaged cortex (43). Inhibition of MMP-2 activity has neuroprotective effects and reduces edema and brain damage (44). The expression level of MMP-2 was positively correlated with the size of CI and neurological deficit score in AMI patients with combined CI, the higher the expression level of MMP-2, the higher the risk of AMI with CI (45). TIMP-1 levels are associated with an increased risk of death and primary disability after acute ischemic stroke, and serum TIMP-1 levels in patients with middle cerebral artery infarction are inversely correlated with survival rates (46, 47). Increased activity as well as increased levels of MMP-2 in atherosclerotic plaques exert a prothrombotic effect by enhancing platelet activation, which increases the incidence of ischemic cerebrovascular events (48).

In the fourth protein cluster, there are four proteins, B2M, CALR, FCGRT, and LRG1. The KEGG pathway enrichment analysis shows that it is majorly enriched in hsa04612: Antigen processing and presentation (FDR = 0.0218). The Reactome pathway enrichment analysis shows that it is mainly enriched in HSA-983170: Antigen Presentation: Folding, assembly, and peptide loading of class I MHC (FDR = 0.0238). In contrast, there was no related pathway enriched by the WikiPathways enrichment analysis. In these proteins, elevated levels of Beta-2-Microglobulin (B2M) may cause neurological impairment resulting in cognitive deficits (49). LRG1 is an important factor in pathogenic angiogenesis, a critical stage in the development of neurological diseases such as stroke (50, 51). LRG1 significantly enhances apoptosis and autophagy during tMCAO, and a positive correlation was shown between LRG1 and severity in patients with cardiogenic embolic stroke (52, 53).

The fifth protein cluster contains a total of eight proteins, namely ADAMTS13, APOA1, APOC1, CP, GIG25, HP, LCAT, and PLTP. The KEGG pathway enrichment analysis shows that it is mainly enriched in hsa04979: cholesterol metabolism (FDR 1.03E-06). The Reactome pathway enrichment analysis shows that it is mainly enriched in HSA-174824: plasma lipoprotein assembly, remodeling, and clearance (FDR = 2.78E-05), HSA-8964058: HDL remodeling (FDR = 2. 78E-05), HSA-382551: transport of small molecules (FDR = 0.0019), HSA-2168880: clearance of heme from plasma (FDR = 0.0066), HSA-2168880: clearance of heme from plasma (FDR = 0.0066), HSA-8963898: plasma lipoprotein assembly (FDR = 0.0111), HSA-8964043: plasma lipoprotein clearance (FDR = 0.0268), and HSA-9029569: NR1H3 and NR1H2 regulate gene expression related to cholesterol transport and efflux (FDR = 0.0292). The WikiPathways enrichment analysis revealed its enrichment in WP430: cholesterol production inhibition by statins (FDR = 3.16E-07), WP5108: familial hyperlipidemia type 1 (FDR = 8.49E-06), WP5109: familial hyperlipidemia type 2 (FDR = 8. 49E-06), WP5110: familial hyperlipidemia type 3 (FDR = 8.49E-06), WP5112: familial hyperlipidemia type 5 (FDR = 8.49E-06), WP5111: familial hyperlipidemia type 4 (FDR = 1. 02E-05), WP3601: lipid particle composition (FDR = 0.00078), WP4522: metabolism of LDL, HDL and TG pathways, including disease (FDR = 0.0019), WP2878: PPAR-alpha pathway (FDR = 0. 0041), WP1533: vitamin B12 metabolism (FDR = 0.013), WP176: folate metabolism (FDR = 0.0208), WP3942: PPAR signaling pathway (FDR = 0.0208), and WP15: selenium micronutrient network 0.0287). Among these proteins, elevated APOA1 levels reduce the risk of ischemic stroke, may delay the progression of atherosclerotic lesions, and also promote lesion regression (54–57). CP can help regulate cellular iron homeostasis. Expression of CP is rapidly upregulated after permanent middle cerebral artery occlusion (pMCAO), while CP deficiency leads to dysregulation of iron homeostasis, increased oxidative damage, increased lesion size, and impaired functional recovery (58). Glycosylated CP may play a key role in neuroprotection (59). HP improves survival, motor function, and brain injury after cerebral ischemia by binding to HMGB1 and regulating macrophage/microglia polarization (60). Furthermore, HP reduces the risk of ischemic stroke by scavenging free hemoglobin and protecting it from iron-induced oxidative damage, inflammatory responses, and consequent atherosclerosis (61). LCAT may have an anti-atherogenic effect, and LCAT deficiency can cause lead to a severe reduction in HDL (62). PLTP promotes phosphatidylserine externalization on the platelet plasma membrane and accelerates adenosine diphosphate (ADP) or collagen-induced platelet aggregation, and its altered expression may influence atherosclerosis (63, 64).

The sixth protein cluster involves seven proteins, namely AHSG, AMBP, APOC3, APOM, C9, ORM1, and TF. Reactome pathway enrichment analysis showed that it was mainly enriched in HSA-114608: platelet degranulation (FDR =0.0213). The KEGG pathway enrichment analysis as well as the WikiPathways enrichment analysis showed no significant enrichment of pathways. AHSG may enhance the inflammatory response of vascular endothelial cells, the formation of macrophage foam cells, and the proliferation of vascular smooth muscle cells and collagen production, leading to the development of atherosclerosis (65). AMBP may be an atherosclerosis-promoting protein, but its downregulation during ischemia is associated with neuroprotective effects (66, 67). APOC3 plays a key role in the progression of atherosclerosis by increasing the risk of ischemic stroke and counteracting the effects of ApoC3, which can substantially reduce the size of atherosclerotic lesions (68, 69). APOM is involved in atherosclerosis, and its changes are associated with recovery from acute ischemic stroke (56, 70). C9 levels may correlate with the degree of brain injury, and the complement system is strongly associated with the development of neuroinflammation (71). Increased expression of ORM1 after cerebral ischemia exacerbates the disruption of the BBB after ischemic stroke (72), and may also contribute to thrombotic susceptibility through an immunothrombotic mechanism (73). In addition to this, ORM 1 upregulation is also associated with neuroinflammation (74). TF contributes to thrombosis and causes the destabilization of atherosclerotic plaques (75, 76). In addition, transferrin expression is higher in men, increases with age, and is upregulated in response to SARS-CoV-2 infection (77).

The seventh protein cluster contained five proteins, CDK2, HIST1H2AE, MEGF8, SELL, and SPDYA, which were not enriched to the relevant pathways after pathway enrichment analysis. Among these proteins, activation of cell cycle protein-dependent kinases (CDKs) such as CDK2 may cause neuronal death after cerebral ischemia (78). MEGF8 is involved in mediating BMP4 signaling and directing the development of trigeminal ganglion (TG) axons, and bone morphogenetic protein (BMP) signaling has emerged as an important regulator of sensory neuron development (79). Increased levels of soluble L-selectin (SELL) may increase the risk of ischemic stroke (80).

In addition, some differential proteins were not in these seven clusters. Among these proteins, overexpression of C4A may cause changes in prefrontal neurological function, leading to cognitive decline (81). Elevated levels of C4A may lead, for example, to Alzheimer's disease, with cognitive dysfunction (82). C4B is significantly correlated with the ratio of total tau, which may affect the neuroprotective effects of APOE (83). HPR and HP are closely linked and it may lead to higher activity (84). Haptoglobin-related proteins bind to Hb and apolipoprotein-L, which not only link HPR to the cholesterol system, but the HPR/apo-L complex also has a specific trypanosomal lysis effect (85). SERPINA3 can attenuate neuronal injury by interfering with granzyme B-mediated neuronal death after cerebral ischemia (86). Presence of SERPINA3N/SERPINA3 aggregates in cortical oligodendrocytes in areas of brain injury (87).

We also used Cytoscape MCODE to screen the four most important protein clusters in the PPI network. The first cluster has seven proteins, including APOC3, PLTP, APOM, HP, APOA1, LCAT, and APOC1, which are mainly enriched in hsa04979: cholesterol metabolism (FDR = 2.83E-22), WP430: cholesterol production inhibition by statins (FDR = 3.64E-21), and hsa-174824: plasma lipoprotein assembly, remodeling, and clearance (FDR = 9.09E-20) pathways. The second cluster has four proteins, including TIMP2, TIMP1, MMP16, and MMP2, which are mainly enriched in WP129: Matrix metalloproteinase (FDR = 2.46E-19), HSA-1592389: activation of matrix metalloproteinase (FDR = 1.65E-18); and HSA-1474228: degradation of extracellular matrix (FDR = 5.44E-16). The third protein cluster has three proteins, including ORM1, AHSG, and AMBP, which are mainly enriched in HSA-114608: platelet degranulation (FDR = 9.36E-12), HSA-381426: regulation of insulin-like growth factor transport and uptake by insulin-like growth factor binding proteins (IGFBPs) (FDR = 9.36E-12), and HSA-76002: platelet activation, signaling, and aggregation (FDR = 9.36E-12) pathways. The fourth protein cluster has three proteins, including C4A, CFB, and C4B, which are mainly enriched in the WP5090: complement system in neuronal development and plasticity (FDR = 4.75E-27), HSA-166658: complement cascade (FDR = 3.76E-26) and WP2806: complement system (FDR = 2.08E-24) passages. This is close to the four protein clusters obtained by STRING screening mentioned earlier, and their enrichment pathways are also consistent. The present study also has shortcomings. To reduce the effects of confounding factors such as age, sex, race, and comorbid diseases, we minimized these effects by including eligible ischemic stroke patients in the study and matching the selection of healthy controls accordingly, but there are still shortcomings due to the small sample size. Although these confounding factors can only be addressed in future studies, our findings are equally informative for future studies.

## 5. Conclusion

In conclusion, by using the DIA technique to detect the serum protein expression profile before and after stroke rehabilitation treatment, 44 differentially expressed proteins were clarified, and 22 upregulated and downregulated proteins were found, respectively. Based on this, the GO and pathway enrichment analysis of DEPs were performed by using bioinformatics methods, and a DEPs PPI network consisting of 44 nodes and 81 edges was also constructed through the STRING website. We identified complement-related pathways, lipoprotein-related functions and effects, thrombosis and hemostasis, coronavirus disease (COVID-19), and inflammatory and immune pathways as the major pathways involved in neurological improvement after ischemic stroke rehabilitation. By the PRM validation, IGHA1, LRG1, IGHV3-64D, and CP may be biomarkers of neurological recovery after stroke.

## Data availability statement

The original contributions presented in the study are publicly available. This data can be found here: ProteomeXchange Consortium, http://www.proteomexchange.org/, PXD036840.

## Ethics statement

The studies involving human participants were reviewed and approved by Medical Ethics Committee of Xiangya Boai Rehabilitation Hospital. The patients/participants provided their written informed consent to participate in this study.

## Author contributions

WH, NZ, and ST conceived and designed the study. WH and PL performed data analysis. WH and ST wrote the paper. All authors contributed to the article and approved the submitted version.
